# Expansion Microscopy of the Enteric Nervous System: A Feasibility Study

**DOI:** 10.3390/cells14181463

**Published:** 2025-09-18

**Authors:** Xin Xu, Wenchuan Zhang, Menachem Hanani

**Affiliations:** 1 Laboratory of Experimental Surgery, Hadassah-Hebrew University Medical Center, Mount Scopus, Jerusalem 91240, Israel; xuxin1418@163.com; 2 Faculty of Medicine, Hebrew University of Jerusalem, Jerusalem 91120, Israel; 3 Department of Neurosurgery, Shanghai Ninth People’s Hospital, School of Medicine, Shanghai Jiao Tong University, Shanghai 200011, China

**Keywords:** expansion microscopy, enteric nervous system, myenteric plexus, glia, neurons

## Abstract

Expansion microscopy (ExM) enables conventional light microscopes to achieve nanoscale resolution by physically enlarging biological specimens. While ExM has been widely applied in neurobiology, it has not been adapted for the enteric nervous system (ENS). Here, we provide a detailed and reproducible protocol for applying ExM to mouse colonic ENS tissue. The procedure includes preparation of the external muscle layers with the myenteric plexus, histochemical staining for NADPH-diaphorase, immunostaining for glial fibrillary acidic protein (GFAP), anchoring of biomolecules, gelation, proteinase K digestion, and isotropic expansion in a swellable polymer matrix. Step-by-step instructions, required reagents, and critical parameters are described to ensure robustness and reproducibility. Using this protocol, tissues expand 3–5-fold, allowing neuronal somata, fibers, and glial cell processes to be clearly visualized by standard brightfield or fluorescence microscopy. The tissue architecture is preserved, with distortion in the X–Y plane of about 7%. This protocol provides a reliable framework for high-resolution structural analysis of the ENS and can be readily adapted to other peripheral tissues.

## 1. Introduction

Light microscopy has been a cornerstone in biological research, allowing researchers to visualize cells, their substructures, and molecular components. However, the diffraction limit of light, which restricts resolution to approximately 200 nm, has been a significant barrier to observing nanometer-scale biological features under light microscopy. To overcome this, various super-resolution techniques have been developed, such as Stimulated Emission Depletion (STED) microscopy and Stochastic Optical Reconstruction Microscopy (STORM) [1,2]. These methods are powerful, but require sophisticated and expensive instrumentation, and their use is limited to fluorescent probes. Expansion microscopy (ExM), first introduced by Chen et al. in 2015, presents a simpler approach for visualizing structures below the diffraction limit by physically expanding the sample [3].

ExM is based on embedding biological tissues in a swellable hydrogel, which enables isotropic expansion of the sample. This process effectively increases the physical distance between molecules, while maintaining their relative positions, thus permitting high-resolution imaging using conventional microscopy. ExM has been applied to various biological systems, such as cultured cells and tissue sections (mostly of the brain) [4,5]. The technique’s ability to achieve 4- to 20-fold expansion of samples has made it particularly valuable in neurobiology, where resolving intracellular and synaptic structures is essential for understanding brain function and disease [6].

The enteric nervous system (ENS), often referred to as the “second brain,” is a complex network of neurons and glial cells embedded in the gastrointestinal (GI) tract. It consists of two networks, the myenteric and submucosal plexuses, and plays a critical role in regulating GI motility, secretion, and blood flow, as well as interacting with the central nervous system [7]. The morphology of the ENS has been well-characterized by light- and electron-microscopy techniques [8,9,10,11,12]. Still, there has been only a small number of super-resolution studies on the ENS, and none are available on the use of ExM for ENS research [13,14]. High-resolution imaging of neural networks within the ENS is crucial for understanding its role in various GI disorders, including inflammatory bowel diseases, irritable bowel syndrome, and diabetic enteropathy [8,15,16].

Early studies on ExM focused primarily on brain tissues, where the relatively low collagen content and soft extracellular matrix (ECM) facilitated isotropic expansion with minimal structural disruption [3]. However, when ExM was extended to peripheral tissues, such as skin and muscle, researchers encountered difficulties due to the higher collagen content and more rigid ECM components in these tissues [17,18,19]. These factors can hinder uniform expansion, potentially leading to tissue deformation and compromising the resolution. To meet this challenge, several ExM protocols were developed to address the specific needs of peripheral tissues. For instance, enzymatic digestion steps, such as the use of collagenase, have been applied to break down the collagen network present in many peripheral tissues [19]. This approach has proven effective in improving the uniformity of tissue expansion in areas rich in collagen-rich tissues.

In the present work, we applied a modified ExM protocol for the ENS in the mouse colon. By relying on proteinase K (ProK) digestion alone, we aimed to achieve tissue expansion while maintaining the native organization of neurons and glial cells. This approach, which emphasizes neural tissue preservation without over-reliance on ECM degradation, marks a novel attempt to extend ExM to the study of the ENS. This study not only demonstrates the utility of ExM in studying the ENS, but also provides a framework for applying this technique to other smooth muscle-containing tissues, such as blood vessels, trachea and urinary bladder. The ability to visualize networks of neurons and glial cells at a high resolution opens new avenues for research on the peripheral nervous system.

## 2. Experimental Design

### 2.1. Overall Study Design

This protocol was designed to establish ExM as a reliable method for the ENS in the mouse colon. The aim was not to reveal new biological features per se, but rather to define the methodological conditions that allow isotropic expansion of ENS tissues while preserving cellular architecture. The study focused on the myenteric plexus, chosen because of its well-defined organization and accessibility after removal of the mucosa and submucosa.

Unlike protocols for collagen-rich tissues, which often require collagenase digestion, our design relied solely on ProK to achieve expansion. This choice was based on the relatively low collagen content within myenteric ganglia, reducing the need for additional enzymatic treatment. By avoiding collagenase, the protocol minimizes the risk of over-digestion, structural distortion, and variability across preparations.

To validate reproducibility, the protocol incorporates multiple biological replicates, independent tissue segments from each colon, and comparative imaging before and after expansion. The workflow integrates both reduced nicotinamide adenine dinucleotide phosphate–diaphorase (NADPH-d) histochemistry (to label neurons) and glial fibrillary acidic protein (GFAP) immunofluorescence (to label enteric glial cells), enabling the assessment of ExM across different cellular components of the ENS.

### 2.2. Animals and Sampling Strategy

Adult Balb/c mice (3–5 months old, weighing 19–23 g, with an approximately equal sex ratio) were used for all experiments. The animals were housed under standard laboratory conditions with free access to food and water and were maintained on a 12 h light/dark cycle. All experimental procedures were approved by the Animal Care and Use Committee of the Hebrew University–Hadassah Medical School, and were performed in accordance with the National Institutes of Health guidelines for the care and use of laboratory animals.

For each staining group (NADPH-diaphorase histochemistry and GFAP immunofluorescence), tissues were obtained from three independent mice. From each animal, three distal colonic segments (approximately 5 mm in length) were excised and processed independently. This design yielded a total of nine samples per staining group, ensuring both biological and technical replicates.

The colon was carefully removed, opened along the mesenteric border, and pinned flat in a dissection dish lined with silicone elastomer. The mucosa and most of the submucosa were removed, leaving preparations of the external muscle layers with the myenteric plexus exposed. These preparations were used for all subsequent staining and expansion steps.

### 2.3. Staining Design

To visualize distinct cellular populations within the ENS, two complementary labeling approaches were employed prior to tissue expansion.

Neuronal labeling was achieved using NADPH-d histochemistry, which selectively stains nitrergic neurons. This method provides robust cytoplasmic staining that clearly delineates neuronal somata and processes under brightfield microscopy. The strong contrast of NADPH-d labeling makes it particularly suitable for assessing changes in neuronal morphology before and after expansion.

Glial labeling was performed by immunofluorescence staining for GFAP, a widely used marker for enteric glial cells. GFAP immunolabeling allows for visualization of the characteristic thin rim of cytoplasm surrounding the large nucleus and the fine glial processes extending into the ganglia. This enabled us to evaluate the ability of ExM to resolve glial structures that are not easily discernible with conventional light microscopy.

Staining for NADPH-d was performed as described previously [20]. NADPH-d and GFAP staining procedures allowed direct comparison of the same tissue before and after expansion under identical imaging conditions, thereby ensuring that structural differences observed were attributable to the expansion process, rather than to variations in staining.

Negative controls were included by omitting the primary antibody during GFAP immunostaining, ensuring the specificity of the fluorescent signal.

### 2.4. Expansion Procedure Design

The following protocol is based on Asano et al., with several modifications [21]. Details on the chemicals and solutions are presented in Table 1.

#### 2.4.1. Anchoring

To ensure covalent linkage of biomolecules to the hydrogel, tissues were incubated overnight in Acryloyl-X, SE (AcX). This anchoring step is critical for maintaining the relative positions of proteins and labeled structures during subsequent digestion and swelling.

#### 2.4.2. Gelation

Following anchoring, samples were embedded in a polyacrylamide-based swellable hydrogel. The gelation mixture was freshly prepared on ice and contained monomers and crosslinkers supplemented with 4-hydroxy-TEMPO (4HT), ammonium persulfate (APS), and TEMED in a defined ratio. Small tissue fragments (~2.5 × 2.5 mm) were flattened under a coverslip during polymerization. This configuration prevents tissue folding or distortion.

#### 2.4.3. Digestion

After gelation, excess gel was trimmed off, and tissues were incubated in a digestion buffer containing ProK at 50 °C overnight. ProK was selected as the sole enzymatic treatment for ENS tissue, avoiding the use of collagenase that is typically recommended for collagen-rich tissues. Preliminary observations showed that collagenase digestion was unnecessary in myenteric plexus preparations, where collagen is sparse within the ganglia, and could even cause structural degradation.

#### 2.4.4. Expansion

Gels were immersed in deionized water and allowed to swell through three sequential 15 min washes. This process enabled isotropic expansion, typically yielding a 3–5-fold linear increase in tissue dimensions.

### 2.5. Imaging and Quantification

To evaluate the effectiveness of the expansion protocol, tissues were imaged both before and after expansion under identical conditions. Maintaining consistent imaging parameters was essential to ensure that observed differences reflected the expansion process rather than technical variability.

#### 2.5.1. Imaging Platforms

NADPH-diaphorase-labeled tissues were visualized using brightfield microscopy with a water immersion 50× objective. This provided clear images of neuronal somata and fibers in both pre- and post-expansion states.

GFAP-immunostained tissues were visualized using widefield fluorescence microscopy with a water immersion 40× objective. Fluorescent signals were collected using standard filter sets for Alexa Fluor 594 and DAPI.

For both modalities, camera exposure, gain, illumination intensity, and acquisition software settings were kept constant between pre- and post-expansion imaging.

#### 2.5.2. Quantification of Expansion Factor

To determine the linear expansion, neuronal dimensions were measured before and after expansion. Specifically, the major and minor neuronal axes were recorded from digital images using calibrated image analysis software ImageJ (v1.8.0, NIH, Bethesda, MD, USA). The expansion factor was calculated as the ratio of the post-expansion to pre-expansion dimension for each axis.

#### 2.5.3. Assessment of Isotropy and Distortion

Expansion isotropy was evaluated by comparing the expansion factors in the X and Y dimensions. Anisotropy was expressed as the percentage difference between the two axes relative to the mean expansion factor.

### 2.6. Reproducibility and Expected Outcomes

To ensure reproducibility, multiple levels of biological and technical replication were incorporated into the protocol. For each staining group, tissues were obtained from three mice, and three distal colonic segments were excised from each animal. This provided nine independent preparations per group, which were processed and analyzed separately. Within each preparation, multiple neuronal somata were measured, enabling assessment of both within-sample and between-sample consistency.

The expansion process yielded highly reproducible results across different animals and tissue segments. Linear expansion factors typically ranged from 3- to 5-fold, with minimal variability between samples. Measurements of neuronal and glial somata consistently demonstrated a proportional increase in both the major and minor axes, confirming that tissue expansion was nearly isotropic. The average anisotropy was approximately 5–7%, in agreement with previously reported ExM studies in other tissue types, indicating that minor deviations in isotropy do not compromise overall structural fidelity [22].

As expected, structures that were poorly resolved in unexpanded preparations became clearly visible after expansion. Neuronal somata exhibited well-defined boundaries, neuronal fibers were readily distinguished, and glial cells displayed their characteristic thin rim of GFAP-labeled cytoplasm and fine processes. These features, which were not resolvable under conventional light microscopy alone, could be systematically visualized after expansion.

## 3. Materials and Equipment

### 3.1. Animals

Adult Balb/c mice (3–5 months old, weighing 19–23 g, with an approximately equal sex ratio) were used. All experimental procedures were approved by the Animal Care and Use Committee of the Hebrew University–Hadassah Medical School and conformed to the National Institutes of Health guidelines for the care and use of laboratory animals.

### 3.2. Reagents and Chemicals

Refer to Table 1.

### 3.3. Antibodies and Staining Reagents

Rabbit anti-GFAP (Dako, Z0334, Glostrup, Denmark), used at 1:250 dilution.

Donkey anti-rabbit IgG Alexa Fluor 594 (Abcam, ab150068, Cambridge, UK), used at 1:300 dilution.

DAPI (Sigma-Aldrich, D9542, St. Louis, MO, USA), used at 1:500 for nuclear staining.

Nitroblue tetrazolium (NBT) (Sigma-Aldrich, N6876), 0.2 mg mL^−1^ for NADPH-diaphorase staining.

NADPH (Sigma-Aldrich, N7505), 0.5 mg mL^−1^ for NADPH-diaphorase staining.

### 3.4. Consumables

Glass-bottom dishes with 13 mm wells (MatTek Corporation, Ashland, MA, USA).

Coverslips (No. 1.5 thickness).

Silicone elastomer-coated dissection dish.

Fine forceps and dissection pins.

### 3.5. Equipment

Brightfield microscope (Leitz, 50× objective, Oberkochen, Germany) for NADPH-d imaging.

Fluorescence microscope (Zeiss Axioskop, Oberkochen, Germany, 40× objective with Alexa Fluor filter sets) for GFAP imaging.

Digital camera (Pixera Penguin 600CL, San Jose, CA, USA) for image acquisition.

Incubator (Memmert IN30, Schwabach, Germany) for gelation at 37 °C and digestion at 50 °C.

Software: ImageJ v1.8.0 (NIH, Bethesda, MD, USA) for image measurement and expansion factor calculation.

## 4. Detailed Procedure

### 4.1. Tissue Preparation and Fixation

Sacrifice adult Balb/c mice (3–5 months old, 19–23 g) by CO_2_ inhalation.

Open the abdominal cavity and carefully remove the colon.

Place the colon immediately into cold Krebs solution (NaCl 118 mM, KCl 4.7 mM, MgSO_4_ 1.2 mM, NaH_2_PO_4_ 1.5 mM, NaHCO_3_ 14.5 mM, glucose 11.5 mM, CaCl_2_ 2.5 mM), bubbled with 95% O_2_/5% CO_2_.

Open the colon along the mesenteric border and pin flat in a silicone elastomer-lined dish.

Remove the mucosa and most of the submucosa, leaving the external muscle layers with the myenteric plexus exposed.

Fix the tissue in 4% paraformaldehyde (PFA, in PBS) for 30 min at room temperature.

Wash three times in PBS, 10 min each.

### 4.2. Staining Procedures

Note: All staining is performed prior to expansion to enable a direct comparison of pre- and post-expansion images.

#### 4.2.1. NADPH-Diaphorase Histochemistry (Neuronal Labeling)

Incubate fixed tissue in 0.3% Triton X-100 in 0.1 M Tris buffer, pH 7.4 for 20 min at 37 °C.

Transfer to reaction solution containing 0.2 mg mL^−1^ NBT and 0.5 mg mL^−1^ NADPH in Tris buffer. Incubate at 37 °C for 30 min.

Stop the reaction by washing twice in PBS.

#### 4.2.2. GFAP Immunofluorescence (Glial Labeling)

Quench autofluorescence in 50 mM NH_4_Cl for 1 h at room temperature. Wash 3 × 10 min in PBS.

Block and permeabilize in 3% BSA + 0.3% Triton X-100 in PBS for 4 h at room temperature.

Incubate with rabbit anti-GFAP antibody (1:250 in PBS + 1% BSA) for 48 h at 4 °C.

Wash 3 × 30 min in PBS.

Incubate with donkey anti-rabbit Alexa Fluor 594 (1:300 in PBS + 1% BSA) and DAPI (1:500) overnight at room temperature in the dark.

Wash 3 × 30 min in PBS.

### 4.3. Anchoring

Cut stained tissue into ~2.5 × 2.5 mm segments.

Incubate overnight at room temperature in 0.1 mg mL^−1^ Acryloyl-X, SE (AcX) in PBS with gentle shaking.

Wash 2 × 15 min in PBS.

### 4.4. Gelation

Prepare the gelling solution fresh according to Table 1 (monomers + crosslinker + 4HT + TEMED + APS, mixed in a 47:1:1:1 ratio), keep on ice.

Immerse tissues in the gelling solution for 30 min at 4 °C in the dark.

Transfer tissue into a 13 mm glass-bottom dish containing 20 µL gelling solution. Flatten tissue gently under a coverslip with a droplet of gelling solution to avoid folding.

Allow polymerization at 37 °C for 2 h.

Remove coverslip and trim off excess gel. Shape asymmetrically to facilitate orientation during later steps.

### 4.5. Digestion

Transfer gels into 2 mL digestion buffer containing ProK (see Table 1).

Incubate overnight at 50 °C.

Note: ProK digestion alone is sufficient for ENS tissue; collagenase is unnecessary and may damage ganglionic structure.

### 4.6. Expansion

Transfer gels into excess deionized water and incubate for 15 min.

Replace with fresh water and repeat for 2 additional cycles (3 × 15 min total).

Monitor expansion by measuring gel dimensions at each step. A 3–5-fold linear increase is typically achieved.

### 4.7. Imaging and Quantification

Image tissues before and after expansion, using the same optics and identical imaging parameters. (NADPH-d samples: brightfield microscopy; GFAP samples: widefield fluorescence microscopy).

Acquire images with identical exposure, illumination, and gain settings.

Measure the major and minor axes of neuronal somata using ImageJ.

Calculate expansion factor as post-expansion/pre-expansion size.

Assess isotropy by comparing expansion factors in X and Y dimensions; calculate anisotropy as percentage deviation.

## 5. Expected Results

Using this protocol, users can expect to achieve a reproducible linear expansion of 3–5-fold in mouse colonic ENS tissues. The expansion process is nearly isotropic, with minor anisotropy of approximately 5–7% in the X–Y plane.

### 5.1. Neuronal Expansion and Size Quantification

**Figure 1:**
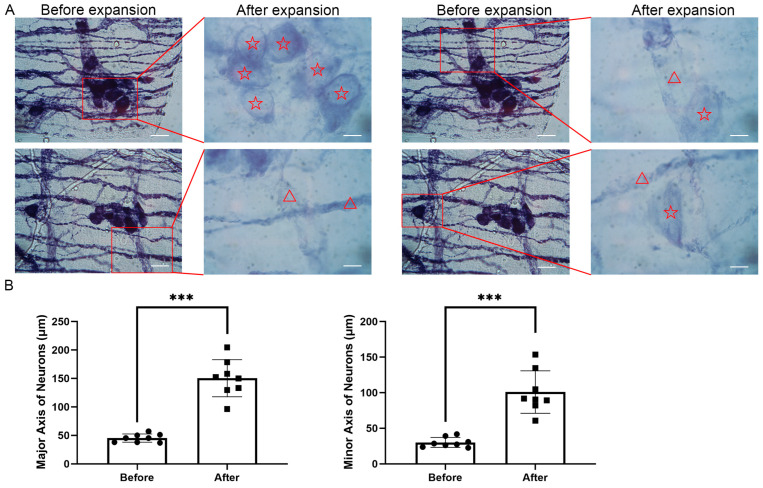
Expansion of neuronal somata and fibers in mouse colonic myenteric plexus. (**A**) Representative images of neuronal somata and fibers in the same tissue before (**left**) and after expansion (**right**). All the images were obtained under the same conditions. The tissue was stained using NADPH-d histochemistry. The boxed areas in the images (**left**) highlight regions shown in detail at the same magnification (**right**). Neuronal somata are marked with red stars (☆), and nerve fibers are indicated with red triangles (Δ). Scale bars, 60 μm. (**B**) Quantification of the size changes in somata before and after expansion. The average major axis increased from 44.4 μm to 154.7 μm after expansion, a factor of 3.48 (*** *p* < 0.001). The average minor axis increased from 29.7 μm to 110.8 μm after expansion, a factor of 3.73 (*** *p* < 0.001). Cell size was measured by ImageJ (v1.8.0). Data are presented as mean ± standard deviation (SD), n = 8 (neuronal cells).

We chose to start with the NADPH-d staining of nerves in the myenteric plexus, because the visibility of the intensely stained cells allowed us to follow the changes in the tissue throughout the procedures (during the procedure, the tissue becomes fully transparent). Starting with the method for intact tissues described by Asano et al. (2018) [21], we tested various conditions until arriving at the protocol described above (see Table 1). We found that a prolonged incubation with ProK at 37 °C was essential, and that digestion by collagenase, which was recommended by Chuang et al. [19], was not required. After expansion, the neuronal somata exhibited about a 3.5-fold increase in size, which helped in resolving single neurons and providing fine details of the nerve bundles and of single nerve fibers in them (Figure 1A). The tissue architecture was preserved, allowing for a clearer visualization of neuronal networks and finer features of the neurons.

To find out whether the expansion was isotropic in the X-Y plane we measured the major and minor axes of the neuronal somata before and after the expansion. As depicted in Figure 1B, the average major axis before expansion was 44.4 μm, and after expansion it increased to 154.7 μm (*p* < 0.001), giving a ratio of 3.48. The minor axis expanded from 29.7 μm to 110.8 μm (*p* < 0.001), giving a ratio of 3.73. Statistical analysis indicated that the difference was highly significant. This ~3.5-fold increase is consistent with the expected expansion factor. We calculated the anisotropy by dividing the difference, 3.73 − 3.48 = 0.25 by 3.605 (the average 3.48 of 3.73), and obtained the value of 6.9%.

### 5.2. Fluorescence Imaging of Enteric Glia

**Figure 2:**
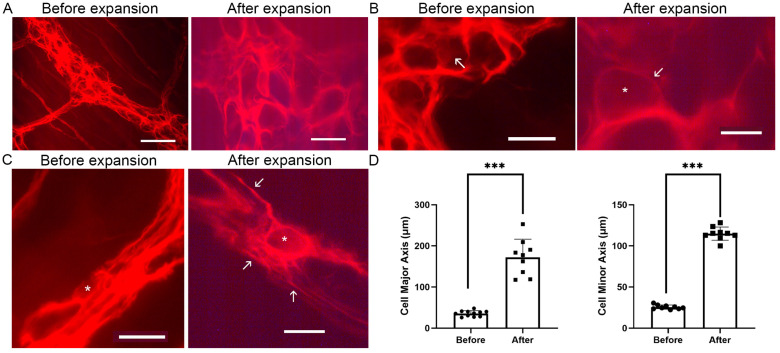
Fluorescence imaging of myenteric plexus in mouse colon, before and after expansion. (**A**) Representative fluorescence images of glia before (**left**) and after (**right**) expansion. The images were obtained under the same conditions. The tissue was immunostained for GFAP. Scale bars, 60 μm. (**B**) Representative images of glial cells within the ganglia, before and after expansion. The cell body of a glial cell is indicated with an arrow before expansion, and arrow and * after expansion. (**C**) Glial cells within the fiber tracts, before and after expansion. The expansion enhanced the visualization of glial cell bodies (*) and processes (arrows). Scale bars for (**B**,**C**), 20 μm. (**D**) Quantification of the size changes in neuronal cell contours (defined by the empty spaces surrounded with labeled glia), before and after expansion. The average major axis increased from 35.6 μm before expansion to 172.2 μm after expansion, a factor of 4.83 (*** *p* < 0.001). The average minor axis increased from 25.6 μm before expansion to 114.9 μm after expansion, a factor of 4.48 (*** *p* < 0.001). Cell size was measured by ImageJ. Data are presented as mean ± SD, n = 9 (neuronal cells contours).

Glial cells in the mouse colonic myenteric plexus were immunostained for GFAP and observed under a fluorescence microscope. Figure 2A shows representative images before and after expansion. Following expansion, the structural features became more distinguishable, with an apparent increase in cell size and the visibility of finer details. The architecture of the tissue remained intact, demonstrating the preservation of cellular relationships.

Figure 2B shows glial cells within the ganglia before and after expansion. The GFAP-labeled cytoplasmic rim, characteristic of enteric glial cells, is clearly visible after expansion. This improved visualization underscores the ability of ExM to resolve fine structural details within the ganglia. Figure 2C shows glial cells in the fiber tracts. Fine glial processes (arrows) are seen only after expansion. The background fluorescence is slightly higher after expansion, apparently because of the much greater tissue thickness.

Figure 2D presents the size of neuronal cell contours before and after expansion. The average major axis of neuronal cell contours before expansion was 35.6 μm, and after expansion it was 172.2 μm, a factor of 4.83 (*p* < 0.001), and the average minor axis increased from 25.6 μm to 114.9 μm, a factor of 4.48 (*p* < 0.001). Anisotropy is as follows: 4.83 − 4.48 = 0.35, divided by 4.655 (the average 4.48 of 4.83) = 7.51%.

These results confirm the utility of ExM in improving structural clarity in fluorescence imaging, making it a powerful tool for further investigations into the architecture of the ENS.

## 6. Comment

In this work, we identified the optimal conditions for applying ExM to the myenteric plexus in the mouse colon. Rather than introducing new structural features of the ENS, our aim was to determine the appropriate methodological approach to expand the ENS while preserving its architecture. This represents a first step in adapting ExM for ENS and visceral organs in general, allowing for future high-resolution studies of peripheral neural networks.

Previous applications of ExM have largely focused on brain tissues, where the sparse ECM and very low collagen content made expansion quite straightforward [22,23,24,25,26]. Peripheral tissues, such as the GI tract, pose challenges for ExM, due to their dense ECM. However, we found that in the ENS, ProK digestion alone proved to be sufficient, without compromising structural integrity. Prior studies on collagen-rich tissues like skin and cornea required enzymatic treatments, such as the use of collagenase to achieve consistent expansion [19]. Myenteric ganglia contain little connective tissue, but the muscle layers surrounding it are rich with collagen [9]. Also, a small amount of submucosal collagen very likely remained adhering to the circular muscle. In spite of that, the use of collagenase was not essential. Still, we obtained a relatively high degree of anisotropy, and for future refinement of the method, the use of this enzyme may be considered.

Results showed that by optimizing the protocol for the ENS, we achieved expansion while preserving the fine details of neuronal and glial structures. Using a collagenase-free approach, we simplified the protocol, reducing the potential for over-digestion and distortion, which can be problematic in collagenase-treated tissues. The successful expansion allowed us to visualize neuronal structures at a scale that was previously unachievable with conventional microscopy. Understanding the structural organization of the ENS is key to unraveling the mechanism’s underlying gut function and its dysregulation.

As shown in Figure 1, ExM is highly suitable for bright field microscopy. In most previous studies on ExM, fluorescence microscopy was used, and in very few cases it was applied for bright field [27], which is available in practically every light microscope.

The GFAP staining of enteric glial cells observed in this study aligns with previous findings that describe a thin rim of GFAP-labeled cytoplasm surrounding the large nucleus [28]. This characteristic morphology, attributed to the limited cytoplasmic content of enteric glia, was clearly visualized in our expanded tissue samples (Figure 2B). The ExM method provided enhanced clarity of glial processes and their spatial relationships within the myenteric plexus. These observations highlight the utility of ExM in corroborating and extending prior structural descriptions of enteric glial cells.

Despite the satisfactory results, several limitations should be pointed out. Although ExM allowed a better visualization of ENS structures, it did not fully resolve the intricate organelle details of the neurons. However, this can be overcome by using confocal microscopy of the expanded tissue. It should be remembered that after expansion, the tissue became about X4 thicker, and obviously confocal microscopy would greatly improve the image quality. As stated above, our aim was to employ conventional microscopy as a proof of principle. Another limitation is some tissue distortion during the expansion process. Quantification of expansion factors in the X and Y directions revealed anisotropy of approximately 7%, which is close to the previously reported value of 5% anisotropy in ExM [22]. Future refinements in expansion protocols may further minimize such distortion.

## Figures and Tables

**Table 1:** Information on expansion microscopy solutions.

	Stock Concentration	Amount	Final Concentration	Supplier	Catalog Number
**Anchoring solution**					
Acryloyl-X SE	10 mg mL^−1^ in DMSO		0.1 mg mL^−1^ in 1× PBS	Tokyo Chemical Industry (Tokyo, Japan)	A3450
**Monomer solution (Stock X)**					
Sodium acrylate	380 mg mL^−1^	2.25 mL	86 mg mL^−1^	Sigma (St. Louis, MO, USA)	408220
Acrylamide	500 mg mL^−1^	0.5 mL	25 mg mL^−1^	Sigma	A9099
N,N′-Methylenebisacrylamide	20 mg mL^−1^	0.75 mL	1.5 mg mL^−1^	Sigma	146072
Sodium chloride	292 mg mL^−1^	4 mL	117 mg mL^−1^	Bio-Lab (Ashkelon, Israel)	855921
PBS 10× stock	10×	1 mL	1×		
Sterile water		0.9 mL		Baxter (Deerfield, IL, USA)	UKF7114
**Gelling solution**					
Stock X		188 μL			
4HT ^†^	5 mg mL^−1^	4 μL	0.1 mg mL^−1^	Sigma	H8258
TEMED ^‡^	100 mg mL^−1^	4 μL	2 mg mL^−1^	Sigma	T7024
APS ^§^	100 mg mL^−1^	4 μL	2 mg mL^−1^	Sigma	A3678
**Digestion buffer**					
Triton X-100	100%	2.5 mL	0.5%	Sigma	T9284
EDTA ^¶^		0.146 g	0.29 mg mL^−1^	BDH chemicals (Mumbai, India)	28025
Tris aqueous solution, pH 8	1 M	25 mL	50 mM	AMRESCO (Solon, OH, USA)	0826
Sodium chloride		23.38 g	46.7 mg mL^−1^	Bio-Lab	855921
Sterile water		Add up to 500 mL		Baxter	UKF7114
**Proteinase K digestion buffer**					
Digestion buffer		2 mL			
Proteinase K	11.38 Units mg^−1^	1.42 mg	8 Units mL^−1^	Fisher Bioreagents (Waltham, MA USA)	BP1700-100

^†^ 4HT, 4-hydroxy-2,2,6,6-tetramethylpiperidin-1-oxyl; ^‡^ TEMED, tetramethylethylenediamine; ^§^ APS, ammonium persulfate; ^¶^ EDTA, ethylenediaminetetraacetic acid.

## Data Availability

The data that support the findings of this study are available from the corresponding authors upon reasonable request.

## Abbreviations

The following abbreviations are used in this manuscript:

4HT	4-hydroxy-2,2,6,6-tetramethylpiperidin-1-oxyl
AcX	Acryloyl-X SE
APS	ammonium persulfate
BSA	bovine serum albumin
ECM	extracellular matrix
EDTA	ethylenediaminetetraacetic acid
ENS	enteric nervous system
ExM	expansion microscopy
GFAP	glial fibrillary acidic protein
GI	gastrointestinal
NADPH-d	reduced nicotinamide adenine dinucleotide phosphate–diaphorase
PBS	phosphate-buffered saline
ProK	Proteinase K
RT	room temperature
SD	standard deviation
STED	Stimulated Emission Depletion
STORM	Stochastic Optical Reconstruction Microscopy
TEMED	tetramethylethylenediamine

## References

[1] Blom H., Widengren J. (2017). Stimulated Emission Depletion Microscopy. Chem. Rev..

[2] Rust M.J., Bates M., Zhuang X. (2006). Sub-diffraction-limit imaging by stochastic optical reconstruction microscopy (STORM). Nat. Methods.

[3] Chen F., Tillberg P.W., Boyden E.S. (2015). Optical imaging. Expansion microscopy. Science.

[4] Bissen D., Kracht M.K., Foss F., Acker-Palmer A. (2022). Expansion microscopy of mouse brain organotypic slice cultures to study protein distribution. STAR Protoc..

[5] Park C.E., Cho Y., Cho I., Jung H., Kim B., Shin J.H., Choi S., Kwon S.K., Hahn Y.K., Chang J.B. (2020). Super-Resolution Three-Dimensional Imaging of Actin Filaments in Cultured Cells and the Brain via Expansion Microscopy. ACS Nano.

[6] Truckenbrodt S., Sommer C., Rizzoli S.O., Danzl J.G. (2019). A practical guide to optimization in X10 expansion microscopy. Nat. Protoc..

[7] Furness J.B. (2012). The enteric nervous system and neurogastroenterology. Nat. Rev. Gastroenterol. Hepatol..

[8] Sharkey K.A., Mawe G.M. (2023). The enteric nervous system. Physiol. Rev..

[9] Gabella G., Johnson L.R. (1987). Structure of Muscles and Nerves in the Gastrointestinal Tract.

[10] Gulbransen B.D., Sharkey K.A. (2012). Novel functional roles for enteric glia in the gastrointestinal tract. Nat. Rev. Gastroenterol. Hepatol..

[11] Phillips R.J., Powley T.L. (2007). Innervation of the gastrointestinal tract: Patterns of aging. Auton. Neurosci..

[12] Furness J.B., Di Natale M., Hunne B., Oparija-Rogenmozere L., Ward S.M., Sasse K.C., Powley T.L., Stebbing M.J., Jaffey D., Fothergill L.J. (2020). The identification of neuronal control pathways supplying effector tissues in the stomach. Cell Tissue Res..

[13] Mercado-Perez A., Hernandez J.P., Fedyshyn Y., Treichel A.J., Joshi V., Kossick K., Betageri K.R., Farrugia G., Druliner B., Beyder A. (2024). Piezo2 interacts with E-cadherin in specialized gastrointestinal epithelial mechanoreceptors. J. Gen. Physiol..

[14] Cao N., Merchant W., Gautron L. (2024). Limited evidence for anatomical contacts between intestinal GLP-1 cells and vagal neurons in male mice. Sci. Rep..

[15] Nguyen T.T., Baumann P., Tüscher O., Schick S., Endres K. (2023). The Aging Enteric Nervous System. Int. J. Mol. Sci..

[16] Holland A.M., Bon-Frauches A.C., Keszthelyi D., Melotte V., Boesmans W. (2021). The enteric nervous system in gastrointestinal disease etiology. Cell. Mol. Life Sci..

[17] Neuman R.E., Logan M.A. (1950). The determination of collagen and elastin in tissues. J. Biol. Chem..

[18] Newsome D.A., Foidart J.M., Hassell J.R., Krachmer J.H., Rodrigues M.M., Katz S.I. (1981). Detection of specific collagen types in normal and keratoconus corneas. Investig. Ophthalmol. Vis. Sci..

[19] Chuang Y.H., Wu Y.F., Lin Y.H., Chen Y.H., Zhou Y.X., Hsu S.C., Lee H.M., Chiang A.S., Chen Y.C., Chen S.J. (2024). Super-Resolution Imaging in Collagen-Abundant Thick Tissues. Small Struct..

[20] Hanani M., Grossman S., Nissan A., Eid A. (2012). Morphological and quantitative study of the myenteric plexus in the human tenia coli. Anat. Rec..

[21] Asano S.M., Gao R., Wassie A.T., Tillberg P.W., Chen F., Boyden E.S. (2018). Expansion Microscopy: Protocols for Imaging Proteins and RNA in Cells and Tissues. Curr. Protoc. Cell Biol..

[22] Tillberg P.W., Chen F., Piatkevich K.D., Zhao Y., Yu C.C., English B.P., Gao L., Martorell A., Suk H.J., Yoshida F. (2016). Protein-retention expansion microscopy of cells and tissues labeled using standard fluorescent proteins and antibodies. Nat. Biotechnol..

[23] Chozinski T.J., Halpern A.R., Okawa H., Kim H.J., Tremel G.J., Wong R.O., Vaughan J.C. (2016). Expansion microscopy with conventional antibodies and fluorescent proteins. Nat. Methods.

[24] Campbell L.A., Pannoni K.E., Savory N.A., Lal D., Farris S. (2021). Protein-retention expansion microscopy for visualizing subcellular organelles in fixed brain tissue. J. Neurosci. Methods.

[25] Sarkar D., Kang J., Wassie A.T., Schroeder M.E., Peng Z., Tarr T.B., Tang A.H., Niederst E.D., Young J.Z., Su H. (2022). Revealing nanostructures in brain tissue via protein decrowding by iterative expansion microscopy. Nat. Biomed. Eng..

[26] Klimas A., Gallagher B.R., Wijesekara P., Fekir S., DiBernardo E.F., Cheng Z., Stolz D.B., Cambi F., Watkins S.C., Brody S.L. (2023). Magnify is a universal molecular anchoring strategy for expansion microscopy. Nat. Biotechnol..

[27] Zhu C., Wang A., Chen L., Guo L., Ye J., Chen Q., Wang Q., Yao G., Xia Q., Cai T. (2021). Measurement of expansion factor and distortion for expansion microscopy using isolated renal glomeruli as landmarks. J. Biophotonics.

[28] McClain J., Grubišić V., Fried D., Gomez-Suarez R.A., Leinninger G.M., Sévigny J., Parpura V., Gulbransen B.D. (2014). Ca^2+^ responses in enteric glia are mediated by connexin-43 hemichannels and modulate colonic transit in mice. Gastroenterology.

